# Increased genitourinary fistula rate after bevacizumab in recurrent cervical cancer patients initially treated with definitive radiochemotherapy and image-guided adaptive brachytherapy

**DOI:** 10.1007/s00066-017-1178-x

**Published:** 2017-07-18

**Authors:** Alina Sturdza, Sandra Hofmann, Marlene Kranawetter, Stephan Polterauer, Christoph Grimm, Michael Krainer, Christian Kirisits, Richard Pötter, Alexander Reinthaller, Richard Schwameis

**Affiliations:** 1 Department of Radiation Oncology, Comprehensive Cancer CenterVienna, Medical University of Vienna, Vienna, Austria; 2 Department of general Gynecology and Gynecologic Oncology, Comprehensive Cancer Center Vienna, Medical University Vienna, Waehringer Strasse 18–20, Vienna, Austria; 3 Clinical Division of Oncology, Department of Medicine 1, Comprehensive Cancer Center, Medical University Vienna, Vienna, Austria; 4 Karl Landsteiner Institute for General Gynecology and Experimental Gynecologic Oncology, Vienna, Austria

**Keywords:** Avastin, Radiotherapy, Chemotherapy, Cervical cancer, Toxicity, Avastin, Strahlentherapie, Chemotherapie, Zervixkarzinom, Toxizität

## Abstract

**Background and purpose:**

Patients with recurrent cervical cancer (RecCC) who received definitive radiochemotherapy including image-guided adaptive brachytherapy (IGABT) as primary treatment are currently treated in our institution with palliative intent by chemotherapy (CHT) combined with bevacizumab (BEV). We aim to evaluate the risk of gastrointestinal (GI)/genitourinary (GU) fistula formation in these patients.

**Materials and methods:**

Data of 35 consecutive patients with RecCC treated initially with radiochemotherapy and IGABT were collected. Known and presumed risk factors associated with fistula formation were evaluated. Fistula rate was compared between patients receiving CHT or CHT+BEV.

**Results:**

Of the 35 patients, 25 received CHT and 10 patients received CHT+BEV. Clinical characteristics were comparable. Fistulae were reported in 6 patients: two fistulae (8%) in the CHT group, four (40%) in the CHT+BEV group. GU fistula occurred in the CHT+BEV group only (3/4). Of these 6 patients with fistulae, 5 (83%) had undergone previous invasive procedures after the diagnosis of RecCC and 1 patient had undergone pelvic re-irradiation; 3/6 patients had developed a local recurrence. No other risk factors for fistula formation were identified.

**Conclusion:**

In patients with RecCC after definitive radiochemotherapy including IGABT, the addition of BEV to CHT may increase the risk for GU fistula formation, particularly after invasive pelvic procedures. Future clinical studies are required to identify predictors for fistula formation to subsequently improve patient selection for the addition of BEV in the RecCC setting.

## Zusammenfassung

**Hintergrund und Ziel:**

Patientinnen mit Rezidiv eines Zervixkarzinoms (RecCC), die primär eine bildgesteuerte adaptive Brachytherapie (IGABT) und kombinierte Radiochemotherapie (RCHT) erhalten hatten, werden derzeit in unserem Institut mit einer Kombination aus Chemotherapie (CHT) und Bevacizumab (BEV) behandelt. Ziel dieser Studie war es, das Risiko für das Auftreten gastrointestinaler (GI) sowie urogenitaler (GU) Fisteln unter CHT sowie CHT + BEV zu analysieren.

**Patienten und Methode:**

In diese retrospektive Datenanalyse wurden insgesamt 35 konsekutive Patientinnen mit RecCC, die primär mit RCHT und IGABT behandelt worden waren, inkludiert. Klinisch-pathologische Risikofaktoren für das Auftreten einer GI- sowie GU-Fistel wurden erhoben. Die Raten an Fistelbildungen in der CHT-Gruppe und in der Gruppe mit CHT + BEV wurden verglichen.

**Ergebnisse:**

Von 35 Patientinnen erhielten 25 eine CHT und 10 CHT + BEV. Insgesamt wurde bei 6 Patientinnen eine Fistel diagnostiziert, davon 2 in der Gruppe mit CHT (8 %) und 4 in der mit CHT + BEV (40 %). GU-Fisteln entwickelten sich ausschließlich in der Gruppe mit CHT + BEV (3/4; 75 %). Von den 6 Patientinnen (83 %) mit Fistelbildung hatten 5 eine vorangehende invasive diagnostische Abklärung und 1 Patientin wurde einer neuerlichen Radiotherapie unterzogen. Ein Lokalrezidiv hatten 3/6 Patientinnen entwickelt. Weitere Risikofaktoren für die Entwicklung einer Fistel konnten nicht identifiziert werden.

**Schlussfolgerung:**

Die zusätzliche Gabe von BEV zur CHT bei RecCC-Patienten, die primär mit IGABT und RCHT behandelt wurden, könnte möglicherweise zu einer erhöhten Rate an GU-Fisteln führen. Zukünftige Studien sollten Risikofaktoren für Fistelbildung untersuchen, um eine gründliche Patientenselektion für die Gabe von BEV bei RecCC zu ermöglichen.

For patients with locally advanced cervical cancer (LACC), concurrent radiochemotherapy including brachytherapy (BT) is the standard therapy, leading to good oncologic results [1–3]. The reported absolute risk of developing grade 3–4 (G3–4) genitourinary (GU) or gastrointestinal (GI) fistulae through definitive radiation therapy including standard BT with dose prescription to point A is around 9% (overall G3–4 toxicity 30%) [4]. Within the last few years, modern radiotherapy techniques have been introduced into the treatment of LACC. These include magnetic resonance (MR) image-guided adaptive brachytherapy (IGABT) [5] with dose prescription individualised to the target at the time of BT. In a recently published cohort of 731 patients, the overall actuarial rate of G3–4 GI and GU toxicity after definitive radiochemotherapy and IGABT was 11% at 5 years [6, 18]. A previous publication from our centre on 156 patients in whom systematic IGABT was performed reported only 2/156 fistula events [5]. Despite excellent overall local tumour control results, several series [5, 7, 8] still observed relatively high rates of distant recurrences after IGABT, especially in patients with advanced stage (stage III/IV) and/or nodal disease [6]. These distant metastases require cytotoxic treatment.

Patients with recurrent cervical cancer (RecCC), depending on the site of relapse (local, distant), have been treated by systemic chemotherapy (CHT), typically with paclitaxel-containing regimes. Since 2014, the use of bevacizumab (BEV) in combination with systemic CHT for the treatment of recurrent or metastatic cervical cancer has become common. It has been shown that the addition of BEV was associated with a significant increase in progression-free and overall survival [9]. However, it has also been reported that CHT+BEV is associated with an increased risk of fistula formation when compared to CHT alone in patients with cervical cancer [10], even in the absence of radiation treatment. In a subset analysis of the Gynecologic Oncology Group trial (GOG 240) published in abstract form [11], it was observed that among the patients who developed GI vaginal fistula, 100% had received prior pelvic radiation, some including standard brachytherapy. However, little is known about the risk factors for fistula formation in the presence of BEV. This study aims to evaluate whether patients with RecCC treated initially by concurrent radiochemotherapy and IGABT have an increased risk for GI and GU fistula formation after CHT+BEV treatment.

## Materials and methods

### Patients

Consecutive patients diagnosed with RecCC who were treated between 2009 and 2014 in our institution were enrolled in this study. Clinical data were obtained using available tumour databases and by electronic chart review. The 2009 International Federation of Gynecology and Obstetrics (FIGO) classification system was used [12]. Eligible patients had recurrence of a formerly locally advanced cervical cancer that had been initially treated by definitive concurrent radiochemotherapy including IGABT in our institution. Patients treated initially with surgery or those with primary metastatic disease were not eligible.

Tumour status was assessed, the presence of GU and GI fistula identified, and survival and follow-up times were recorded. Risk factors for fistula development including comorbidities (diabetes, arterial hypertension, peripheral vascular disease, thromboembolic events), previously performed bowel surgery, minor interventions/biopsy/major pelvic surgery after RecCC and number of treatment cycles were documented.

The primary endpoint was to document the frequency of fistula events in both groups (CHT or CHT+BEV) in this mono-institutional series.

The Ethics Committee of our institution gave approval prior to initiation of the study (IRB approval number: 1996/2015). All patients consented to treatment according to institutional guidelines, as well as to anonymized assessments and analysis of data and outcome of therapy. Records were anonymized and de-identified prior to analysis.

### Treatment

#### Initial treatment

All patients received definitive concurrent radiochemotherapy. Radiotherapy consisted of external beam radiotherapy (EBRT) concurrent with weekly cisplatin-based CHT (usually cisplatin 5 × 40 mg/m^2^ body surface area) and IGABT. The maximally allowed duration of radiation therapy was 50 days in total.

#### External beam radiotherapy

EBRT was delivered using three-dimensional conformal techniques or intensity-modulated radiation therapy. The clinical target volume (CTV) irradiated through EBRT consisted of the tumour, entire uterus, bilateral parametria, upper vagina (if no vaginal involvement had been present) and the pelvic lymph nodes. In case of lymph node involvement of the common iliac or para-aortic (PAN) lymph nodes (as diagnosed by positron-emission tomography/CT [PET-CT] or laparoscopic staging lymphadenectomy), para-aortic radiotherapy was performed. The prescribed dose was 45 Gy at 1.8 Gy per fraction. Grossly involved lymph nodes, if not surgically removed, were treated with an additional boost (range 55–60 Gy).

#### Image-guided adaptive brachytherapy

Systematically, MRI-based IGABT was performed in all patients diagnosed with LACC with definitive intent. The high-risk CTV (HRCTV) and/or the intermediate-risk CTV (IRCTV) was contoured according to Gyn GEC-ESTRO Recommendations I [13]. Organs at risk (OARs) were contoured: rectum, sigmoid colon and urinary bladder. Dose–volume histogram (DVH) parameters for the HRCTV, IRCTV and OARs were calculated and reported according to Gyn GEC-ESTRO Recommendations II [14]. Dose prescription for target and dose constraints for OARs were applied according to our institutional guidelines [5]. Our planning aim was ≥85 Gy to 90% of the HRCTV (D_90_). This total EQD2 (equivalent dose in 2 Gy per fraction) from EBRT and BT was calculated using an a/β of 10 Gy for tumour (EQD2_10_) and 3 Gy for OARs (EQD2_3_) [15].

### Follow-up

All patients were followed-up in a joint programme by a gynaecologic oncologist and a radiation oncologist specialised in gynaecologic malignancies. Regular follow-up included clinical examination, blood analysis and imaging. Complete remission after initial treatment was defined as the absence of disease in the cervix (uterus), upper vagina, parametrium and regional lymph nodes, as verified by clinical examination, abdominal and thoracic imaging, and biopsy as appropriate. Patients were followed-up every 3 to 4 months for the first 3 years, every 6 months for the following 2 years and annually up to 10 years and beyond thereafter. Follow-up was performed at our institution using standardized questionnaires and assessment forms.

If recurrent disease was suspected, restaging by CT scans of the thorax and abdomen as well as MRI of the pelvis was performed. Whenever possible, recurrent disease was confirmed by biopsy and PET-CT was additionally performed.

### Recurrence treatment

#### Chemotherapy/bevacizumab

Within this study, all patients were treated by either CHT or CHT+BEV. In addition, salvage surgery was performed in selected cases. CHT consisted of paclitaxel in combination with cisplatin (*n* = 18), with carboplatin (*n* = 2), or with topotecan (*n* = 5). Alternatively, selected patients received cisplatin in combination with topotecan (*n* = 9) or carboplatin with docetaxel (*n* = 1). CHT was administered until progression, complete response or treatment-limiting toxicity occurred. Starting 2013, most patients received BEV in addition to CHT (*n* = 10). Thereafter, patients were again followed-up according to the institution’s follow-up program. In a few cases, salvage surgery or radiotherapy of metastases was performed in the presence of oligometastases in lung, liver or periaortic nodes.

### Statistical analysis

Time-to-event analyses were computed using the Kaplan–Meier method and log-rank test, with and IBM SPSS 23.0 for MAC (SPSS 23.0, IBM Inc., Armonk, NY, USA). Time intervals for survival analyses were calculated from the date of biopsy to the date of event or last follow-up. Patients lost to follow-up were censored at the time of last follow-up.

Descriptive statistic values are given as mean (standard deviation, SD). *T*-tests and χ^2^ tests were used to compare patients’ characteristics and risk factors for fistula formation between groups. *P*-values <0.05 were considered statistically significant. Overall survival was analysed using the log-rank test.

## Results

A total of 35 patients were included in this study. Patients’ and treatment characteristics are shown in Table 1. There were no fistulae at the time of relapse diagnostic. CHT was received by 25 patients and 10 patients received CHT+BEV. Patient characteristics and treatment modalities in the both groups were similar. All 10 patients in the CHT +BEV group had undergone interstitial IGABT as opposed to 21/25 in the CHT group (*p* = 0.18).

**Table 1:** Patient characteristics for the whole group (*N* = 35) and broken down by subsequent treatment, i. e. chemotherapy only or chemotherapy plus bevacizumab

Variable	*N* total (%)	CHT (*N* = 25) *N* (%)	CHT+BEV (*N* = 10) *N* (%)
**Initial stage**			
IB	4 (11)	3 (12)	1 (10)
IIB	18 (52)	15 (60)	3 (30)
IIIA–IIIB	8 (22)	4 (16)	4 (40)
IVA	3 (9)	3 (12)	0
IVB	2 (6)	0	2 (20)
**Histology**			
Squamous cell carcinoma	27 (77)	20 (80)	7 (70)
Adenocarcinoma or adeno-squamous	8 (23)	5 (20)	3 (30)
**Grade**			
G1	3 (9)	2 (8)	1 (10)
G2	14 (40)	9 (36)	5 (50)
G3	13 (37)	10 (40)	3 (30)
Not evaluated	5 (14)	4 (16)	1 (10)
**Median age (years)**	51 (32–78)	53 (32–78)	49 (32–64)
**ECOG**			
0	21 (60)	14 (56)	7 (70)
1	12 (34)	10 (40)	2 (20)
2	2 (6)	2 (4)	1 (10)
**Comorbidities**			
*Diabetes*			
Yes	2 (6)	1 (4)	1 (10)
No	33 (94)	24 (96)	9 (90)
*Hypertension*			
Yes	12 (34)	7 (28)	5 (50)
No	23 (66)	18 (72)	5 (50)
*GI surgery*			
Yes	2 appendectomy, 3 bowel resection (14)	4 (16)	1 bowel resection (10)
No	30 (86)	21 (84)	9 (90)
*Thromboembolic event*			
Yes	6 (17)	2 (8)	4 (40)
No	29 (83)	23 (92)	6 (60)
**Primary treatment**	–	CHT	CHT+BEV
EBRT dose (Gy)	45	45	45
EBRT pelvis (*n*)	20	15	5
EBRT extended-field (*n*)	15	10	5
EBRT IMRT/VMAT (*n*)	6 (17)	0	6 (17)
EBRT 3D conformal (*n*)	29 (83)	29 (83)	6 (17)
BT IC only (*n*)	4 (14)	4 (16)	0
BT IC/interstitial	31 (86)	21 (84)	10 (100)
**Surgical LN staging**			
Yes	26 (74)	20 (80)	6 (60)
**Response rate at initial treatment**			
Complete	25 (71)	18 (72)	7 (70)
Partial	10 (29)	7 (28)	3 (30)
**Type of recurrence**			
Local/pelvic	7 (20)	5 (20)	2 (20)
Distal	20 (57)	13 (52)	7 (70)
Combination	8 (23)	7 (28)	1 (10)
**Pelvic minor intervention/surgery/biopsy after RecCC**			
Yes	17 (49)	12 (48)	5 (50)
No	18 (51)	13 (52)	5 (50)
**Response rate (to treatment for recurrence)**			
Complete	7 (20)	5 (20)	2 (20)
Partial	5 (14.3)	2 (8)	3 (30)
Stable disease	4 (11.4)	3 (12)	1 (10)
Progressive disease	15 (42.9)	11 (44)	4 (40)
Unknown	4 (11.4)	4 (16)	0
Median follow-up from recurrence (months)	11 (0–50)	11 (0–50)	11 (5–28)
Median follow-up from recurrence to event (months)	8 (2–27)	11 (2–20)	8 (5–27)
Median follow-up from diagnosis (months)	26 (5–87)	22 (5–87)	38 (15–48)
Alive with no evidence of disease	5 (14.3)	4 (16)	1 (10)
Alive with stable disease	7 (20.0)	4 (16)	3 (30)
Alive with progressive disease	6 (17.1)	4 (16)	2 (20)
Died intercurrently	1 (2.9)	0	1 (10)
Died of cancer	16 (45.7)	13 (52)	3 (30)

*CHT *chemotherapy,* BEV *bevacizumab, *ECOG* Eastern Cooperative Oncology Group, *GI* gastrointestinal, *EBRT* external beam radiotherapy, *IMRT* intensity-modulated radiotherapy, *VMAT* volumetric-modulated arc therapy, *LN* lymph node, *RecCC* recurrent cervical cancer, *BT IC* intracavitary only brachytherapy

Table 2 provides treatment details of the therapy at first diagnosis and the number of events analysed according to the type of treatment at recurrence. No statistically significant difference in radiation dose (90.4 Gy vs. 89.4 Gy, *p* = 0.353) or number of CHT cycles at RecCC (5.1 vs. 6.4, *p* = 0.403) was observed between treatment groups.

**Table 2:** Treatment characteristics at the time of recurrence, dose–volume parameters (DVH) at the time of the initial definitive treatment and number of events based on the type of treatment for recurrent disease

Variable	CHT (*n* = 25)	CHT +BEV (*n* = 10)
*Fistulae*		
Total	2 (8%)	4 (40%)
Rectovaginal	2	1
Vesicovaginal	0	3
*Mean (SD) no. of treatment cycles*		
CHT cycles	5.1 (1.9)	6.4 (4.5)
BEV cycles	–	5.4 (3.6)
*BT application type*		
IC	4	0
IC/IS	21	10
*Radiation dose (Gy)*		
Mean D_90_ HRCTV	90.4 (7.6)	89.4 (5.7)
Mean D_0.1cc_ rectum	73.5 (11.3)	73.1 (8.5)
Mean D_2cc_ rectum	63.6 (7.7)	63.4 (6.9)
Mean D_0.1cc_ sigmoid	78.0 (13.9)	80.0 (7.2)
Mean D_2cc_ sigmoid	64.7 (7.8)	66.7 (5.8)
Mean D_0.1cc_ bladder	100.3 (13.3)	100.5 (7.7)
Mean D_2cc_ bladder	79.8 (8.4)	82.4 (6.0)
Mean D_0.1cc_ bowel	58.5 (29.5)	65.4 (37.4)
Mean D_2cc_ bowel	52.6 (26.3)	55.1 (30.1)

*CHT *chemotherapy,* BEV *bevacizumab,* BT* brachytherapy, *HRCTV *high-risk clinical target volume, *D90* dose to 90% of the HRCTV, *SD* standard deviation, *IC* intracavitary only, *IC/IS* intracavitary and interstitial brachytherapy, *D2cc* dose to 2cc volume of the respective organ (i.e: rectum, sigmoid, bowel, bladder)

A total of six fistulae were recorded (patient tumour and treatment details in Table 3: P1-6). In the CHT+BEV group, a significantly higher rate of fistula formation (4/10 patients, 40%) was observed compared to the CHT only group (2/25 patients, 8%; *p* = 0.043).

**Table 3:** Tumour and treatment characteristics of the patients with events (fistula formation)

Patient no. (P1–6)	FIGO stage	N status	Tumour width at BT (mm)	Applicator type at BT	D_90_ HRCTV (Gy)	D_0.1cc_ rectum (Gy)	D_2cc_ rectum (Gy)	D_0.1cc_ bladder (Gy)	D_2cc_ bladder (Gy)	Complete remission after definitive treatment	Type of first recurrence	Recurrence therapy	Type of fistula (grade)	Biopsy cervix interventions or pelvis surgery after recurrence	Type of intervention
1	IIIB	pN0	80	Interstitial	88.4	73	61	111	92	No	Persistent local disease	CHT + BEV	G2 vesicovaginal	Yes	Repeated ureter stenting Nephrectomy
2	IIB	pN0	60	Interstitial	96	79	69	95	84	Yes	Systemic	CHT + BEV	G3 rectovaginal	Yes	Major pelvic surgery Nephrectomy
3	IIIB	pN1	50	Interstitial	93	74	66	91	75	Yes	Local	CHT	G3 rectovaginal	No	Pelvic re-irradiation
4	IIIB	cN1	50	Interstitial	93	79	70	106	85	No	Persistent local + systemic disease	CHT + BEV	G2 vesicovaginal	Yes	Repeated ureter stenting
5	IIB	cN1	40	Interstitial	84.3	85	70	106	83	Yes	Systemic	CHT	G3 rectovaginal	Yes	Biopsy posterior vaginal wall
6	IIIB	pN1	35	Interstitial	97.6	79	64	98	75	Yes	Systemic	CHT + BEV	G3 vesicovaginal	Yes	Repeated ureter stenting

*CHT *chemotherapy,* BEV *bevacizumab,* BT* brachytherapy, *HRCTV* high-risk clinical target volume, *FIGO *Federation of Gynecology and Obstetrics, *D90* dose to 90% of the HRCTV, *D0.1cc* dose to 0.1cc volume of the respective organ (i.e: rectum, sigmoid, bowel, bladder), *D2cc* dose to 2cc volume of the respective organ (i.e: rectum, sigmoid, bowel, bladder)

GU fistula formation was noticed only in the CHT+BEV group (3/10 patients), of which two were grade 2 and one was grade 3 requiring surgical intervention [16]. Additionally, a grade 3 rectovaginal fistula was documented in this group (P2). In the CHT only group, both events were rectovaginal fistula grade 3. For all patients with G3 rectovaginal fistula a functional colostomy was performed. For the G3 GU fistula, an ileal conduit was necessary.

At the time of recurrence, 2 patients had persistent local disease (incomplete remission), 1 patient had local recurrence (which was treated with palliative re-irradiation) and 3 patients had systemic recurrence (Table 3).

All 6 patients with fistula formation had undergone an invasive procedure or palliative pelvic radiation prior to or during the systemic palliative treatment (Table 3). In the CHT group the events occurred subsequent to a posterior vaginal wall biopsy (P5) and after pelvic re-irradiation (P3). In this group, a further 10 of the remaining 23 patients had undergone an invasive procedure but did not develop fistula (Table 1). In the CHT+BEV group, 4/4 patients with events (100%) had undergone at least a minimally invasive procedure (i. e. ureter stenting) and only 1/6 patients (17%) did not develop an event after an invasive procedure.

We found no association between radiation dose to the target or the reported DVH parameters for OARs and the rate of fistula (Table 4).

**Table 4:** Mean radiation dose for HRCTV and OARs according to fistula status (dose – volume parameters [DVH] patients and Gy)

Variable	Fistula	No fistula	*p*-value
CHT (absolute number of cycles)	2	23	0.043^a^
CHT + BEV (absolute number of cycles)	4	6	–
Mean HRCTV D_90_ (%)	92.1 (5.0)	89.7 (7.4)	0.353^b^
Mean D_0.1cc_ rectum (%)	78.0 (4.6)	72.4 (11.1)	0.060^b^
Mean D_2cc_ rectum (%)	62.8 (7.8)	66.9 (3.6)	0.066^b^
Mean D_0.1cc_ bladder (%)	100.2 (12.6)	101.2 (7.9)	0.806^b^
Mean D_2cc_ bladder (%)	82.1 (6.7)	80.2 (8.1)	0.551^b^
Mean D_0.1_ sigmoid (%)	69.3 (12.5)	80.5 (11.6)	0.084^b^
Mean D_2cc_ sigmoid (%)	59.4 (8.8)	66.5 (6.4)	0.108^b^
Mean D_0.1_ bowel (%)	69.2 (20)	57 (16)	0.193^b^
Mean D_2cc_ bowel (%)	58 (9)	53 (10)	0.278^b^
No. of CHT cycles (%)	5.2 (2.2)	5.6 (3.0)	0.715^b^
No. of BEV cycles (%)	3.8 (1.7)	6.5 (4.3)	0.200^b^

*CHT *chemotherapy,* BEV *bevacizumab,* HRCTV* high-risk clinical target volume, *D90* dose to 90% of the HRCTV, *OAR* organ at risk, *D0.1cc* dose to 0.1cc volume of the respective organ (i.e: rectum, sigmoid, bowel, bladder), *D2cc* dose to 2cc volume of the respective organ (i.e: rectum, sigmoid, bowel, bladder)

^a^χ^2^ test

^b^ *t*-test

Within the cohort, median follow-up was 11 months (range 0–50 months) and overall survival time from the date of diagnosis of RecCC was 8 months (2–27 months) and 26 months (5–87 months) from diagnosis of primary disease. Fistula formation had no significant impact on overall survival (*p* = 0.317).

## Discussion

In this study, patients receiving a combination of CHT and BEV for the treatment of RecCC had a significantly higher risk for fistula formation than patients receiving CHT alone (40 vs. 8%). We observed a notably higher rate of GU fistula in patients treated with CHT+BEV (30% vs. none). The rate of rectovaginal fistula was similar in the two groups (10 vs. 8%).

Previously published studies reported an overall fistula (>grade 2) formation rate of 8.6% (GI vaginal) and 3.2% GI perforation in 218 patients with preliminary metastatic or recurrent cervical cancer treated in the CHT+BEV arm [17] of the GOG 240 study (first data freeze). Furthermore, the rate of fistula (any grade) formation was even higher (12.6%) during CHT+BEV treatment if only patients with prior radiochemotherapy were considered [11]. Overall, GOG 240 reported an increased incidence of 6% of fistula formation (second data freeze) [9]. It is not clear how many patients had received definitive radiochemotherapy including IGABT in the GOG 240 cohort. In our cohort, only patients who received definite IGABT plus radiochemotherapy as initial treatment were included. Therefore, our cohort is the first one comparing CHT and CHT+BEV in LACC patients previously treated with definitive radiochemotherapy and IGABT.

All patients who developed fistulae in our cohort had undergone a minor intervention (5/6, 83%; repeated ureter stenting, biopsy or pelvic re-irradiation) or a major pelvic surgery (1/6, 17%). This is in accordance with the observation of Feddock el al. [19], who associated pelvic radiation and invasive procedures with 8.2% fistula formation, even in the absence of CHT ± BEV. In the CHT group, 43% (10/23) of the non-event patients had undergone an intervention, while in the CHT+BEV only group, 17% (1/6) had undergone an invasive pelvic procedure and did not develop a fistula. This may suggest that BEV in the presence of an invasive pelvic procedure may trigger GU fistula formation. There are case reports in literature suggesting that a combination of perioperative BEV administration and an invasive procedure could result in fistula formation. Aortooesophageal fistula rupture or colovesical fistula during treatment including BEV due to metastatic colorectal cancer have been reported in case studies [25]. One other report describes two late-onset pulmonary fistulae after resection of pulmonary metastasis from colorectal cancer following perioperative CHT with BEV [26]. An increased risk of bowel perforation or fistula formation for patients with recurrent epithelial ovarian cancer treated with BEV has been observed [24, 27, 28].

Although not statistically significant due to the limited number of patients and events, one may observe the fact that 3/6 patients with fistulae had a local recurrence (2 patients never achieved complete local remission and 1 had a local recurrence).

We could not detect any association between comorbidities, radiation dose, performance status or previously performed bowel surgery prior to the diagnosis of RecCC and a higher rate of fistula formation. Our findings are in accordance with previously published reports [20]. In a study including 30 patients with FIGO stage IVA (with bladder or/and rectal infiltration) undergoing radiochemotherapy, the 5‑year fistula-free survival rate was 64%, but no prognostic variables were identified. Although all the patients in the CHT +BEV group underwent interstitial IGABT as opposed to 84% in the CHT group, the use of interstitial implants is not statistically correlated to the occurrence of fistula when BEV is added to the treatment of RecCC (*p* = 0.18). Moreover, recent literature shows that primarily use of advanced IGABT, especially interstitial implants, does not increase late toxicity when compared to intracavitary therapy only [18].

Recent studies suggest that only intermediate- and high-risk RecCC patients show a survival benefit when receiving CHT+BEV [21]. These patients have 2–3 (intermediate-) and 4–5 (high-risk) risk factors from: black race, performance status 1, pelvic disease, prior cisplatin and progression-free interval <365 days. Hence, patient selection is crucial with respect to side effects and complications. Several approaches were performed to identify biomarkers to predict the effect of BEV on an individual basis [22]. However, no predictive biomarker has been established thus far.

CHT+BEV is administered to RecCC patients without curative intent. The quality of life in this palliative setting is of particular importance and should have a substantial influence on the selection of therapy. Interestingly, while GOG 240 reported an increased rate of fistula in the CHT+BEV group, the quality of life was not decreased in this group. Other reports show that fistula formation leads to a substantial reduction in quality of life [23]. Therefore, patient-reported outcomes, as used in GOG 240, might not be appropriate to determine the detrimental effect of fistula formation on quality of life.

Based on our observations, we suggest that when facing persistent disease or local recurrence, with or without systemic disease, caution should be used in prescribing CHT+BEV. This combination should be offered only after thoroughly informing the patients about the high probability of developing fistula, especially GU fistula. Prudence should also be used when facing ureteral stent change or other pelvic interventions, which are common in patients with RecCC.

Admittedly, the shortcomings of this study have to be considered. This was a retrospective data analysis of a small patient cohort from a single institution. The limited number of patients included in this report impaired statistical analysis and multivariate models were not feasible. However, given the lack of published data and increasing number of patients who are possibly candidates for this treatment, we want to raise awareness about the potential risks of using BEV in addition to CHT in the setting of pelvic RecCC and related interventions.

## Conclusion

Until larger studies are available, based on our observations in this small cohort, and given the overall survival benefit [9], CHT+BEV should probably be offered mainly to patients with local control and systemic recurrences. Future clinical studies are required to identify predictive and prognostic factors for fistula formation, in order to improve patient selection for CHT+BEV in the setting of RecCC.
